# Manuka Honey: A Potent Cariostatic Agent— An *in vitro* Study

**DOI:** 10.5005/jp-journals-10005-1494

**Published:** 2018-04-01

**Authors:** Javaregowda P Beena, Punyatoya Sahoo, Sapna Konde, N Sunil Raj, Narayana C Kumar, Manisha Agarwal

**Affiliations:** 1 Reader, Department of Pedodontics and Preventive Dentistry, AECS Maaruti College of Dental Sciences & Research Center Bengaluru, Karnataka, India; 2 Postgraduate Student, Department of Pedodontics and Preventive Dentistry, AECS Maaruti College of Dental Sciences & Research Center Bengaluru, Karnataka, India; 3 Professor and Head, Department of Pedodontics and Preventive Dentistry, AECS Maaruti College of Dental Sciences & Research Center Bengaluru, Karnataka, India; 4 Professor, Department of Pedodontics and Preventive Dentistry, AECS Maaruti College of Dental Sciences & Research Center Bengaluru, Karnataka, India; 5 Professor, Department of Pedodontics and Preventive Dentistry, AECS Maaruti College of Dental Sciences & Research Center Bengaluru, Karnataka, India; 6 Reader, Department of Pedodontics and Preventive Dentistry, AECS Maaruti College of Dental Sciences & Research Center Bengaluru, Karnataka, India

**Keywords:** Antibacterial, Dabur honey, *Lactobacillus*, Manuka honey, *Streptococcus mutans.*

## Abstract

**Aim:**

The aim of the study was to test the antibacterial activity of manuka honey and compare its efficacy with another commercially available honey (Dabur honey) on the cariogenic bacteria on *Streptococcus mutans* and *Lactobacillus.*

**Materials and methods:**

An *in vitro* study was carried out on 40 agar specimens; the samples were divided into two groups of 20 samples consisting of S. *mutans* and *Lactobacil-lus* respectively. The 20 samples in each group were further subdivided into four groups of five each, which were tested with 25% manuka honey, 100% manuka honey, 25% Dabur honey, and 100% Dabur honey for both *Lactobacillus* and *S. mutans* groups. The antibacterial activity was tested using the agar well diffusion method against *S. mutans* and *Lacto-bacillus.* Antibacterial activity was assessed by measuring the diameter of inhibition of zones surrounding the wells. The results obtained were statistically analyzed (one-way analysis of variance test, p-value).

**Results:**

The results showed that 25% of manuka honey has statistically significant (p ≤ 0.001) antibacterial effect than 25% of Dabur honey on both *Streptococcus* and *Lactobacillus* species, and manuka honey with 100% concentration showed a statistically significant (p ≤ 0.001) antibacterial effect than 100% Dabur honey on the same species of bacteria. 100% of both the honeys showed statistically significant (p ≤ 0.001) antibacterial effect than 25% concentrations of the same on *S. mutans* and *Lactobacillus.*

**Conclusion:**

Manuka honey had more antibacterial activity than Dabur honey on *S. mutans* and *Lactobacillus* bacteria in the *in vitro* study. This effect was dependent on the concentration of honey used.

**How to cite this article:** Beena JP, Sahoo P, Konde S, Raj SN, Kumar NC, Agarwal M. Manuka Honey: A Potent Cariostatic Agent—An *in vitro* Study. Int J Clin Pediatr Dent 2018;11(2):105-109.

## INTRODUCTION

Honey has been used since the time of the ancient Egyptians, the Hebrew kingdoms, and historically in China, India, Greece, Rome, and many other nations.^1^ It is considered as an alternative medicine due to many health benefits attributed to it.^2^ Emphasis in this section is placed on the antibacterial properties of honey. Honey is produced by bees from the nectars they collect from flowers. When a bee collects nectar from flowers, it secretes into it enzymes from its pharyngeal gland. There are generally two varieties of honey, monofloral and polyfloral. Monofloral means it is sourced from one species of flora. The honey which is produced from the pollen and nectar from several species of flora is known as polyfloral honey.^3^

The antibacterial property of honey was first recognized in 1892 by Van Ketel. It has often been assumed that this is due entirely to the osmotic effect of its high sugar content. The fact that the antibacterial properties of honey increased when diluted was clearly observed and reported in 1919.^4^ The explanation for this apparent paradox came from the finding that honey contains an enzyme that produces hydrogen peroxide when diluted. This agent was referred to as “inhibine” prior to its identification as hydrogen peroxide.^5^

The research has proven that honey not only aids in inhibiting the growth of dental plaque bacteria but also significantly reduces the amount of acid produced. More specifically, it hinders the bacteria from producing dextran.^6^

Numerous studies have reported comparison of different varieties of honey, such as wildflower honey which are produced from the pollen and nectar of several species of flora, and the honey has an inhibitory effect on around 60 species of bacteria including Grams, aerobes, and anaerobes. A wide range of antifungal activity has also been observed including some species of yeast, aspergil-lus, and common dermatophytes.^7^

Numerous varieties of honey are produced all over the world and the medical properties of each of these reflect the particular floral source native to that place. Manuka honey is made by bees. The bees collect pollen from the flowers of *Leptospermum scoparium* tree. The honey cannot be called as such unless at least 70% of pollen it is made of comes from the manuka tree. The Aboriginal people have harnessed the benefits of the manuka honey for centuries. They grow wild in most parts of New Zealand but are especially seen in coastal areas.^8^

The manuka flower only blooms for a period of 6 weeks in spring season^9^ and is often region-specific.^10,11^ About 70% of manuka honey is of simple sugars like glucose and fructose and the rest is comprised of complex carbohydrates.^12^ However, the water composition of manuka honey is similar to other varieties of honey.

One factor that makes manuka honey stand out is its compound called methylglyoxal, which is quintessential for its antibacterial, antifungal, antimicrobial, and antiseptic properties. The levels of methylglyoxal present in manuka honey are considered a measure of its potency and purity. Hence, a unique manuka factor (UMF) rating system was developed to measure the level of methyl-glyoxal, and higher the level, higher the rating.^13^ Manuka honey with a rating below 10 is considered as effective as any regular honey.

Hence, the aim of the study was to test the antibacterial activity of manuka honey and compare its efficacy with another commercially available honey on the cariogenic bacteria.

## MATERIALS AND METHODS

An *in vitro* study was carried out on 40 agar specimens; the samples were divided into two groups of 20 samples consisting of *S. mutans* and *Lactobacillus* respectively. The 20 samples in each group were further subdivided into four groups of five each, which were tested with 25% manuka honey, 100% manuka honey, 25% commercially available honey, and 100% commercially available honey for both specimens.

### Assay of Antibacterial Activity

The honey samples were tested for their antibacterial activity, according to the agar well-diffusion method proposed by the Clinical and Laboratory Standards Institute (CLSI) against the two reference strains: (1) *S. mutans* and (2) *Lactobacillus* species. The above bacteria were grown (100 mL) in trypticase soy broth at 37°C for 18 hours.

The honey concentrations (w/v) were prepared in sterile saline solution, 25, 100%, and their antibacterial activity was evaluated against the bacterial strains. Thus, 100 mL aliquot of honey dilution was added to each well of blood agar inoculated with bacterial concentration which is similar to 0.5 McFarland tube and incubated at 37°C for 24 hours.

Antibacterial activity was assessed by measuring the diameter of the inhibition zones surrounding the wells. Control plates were prepared with no honey added. All assays were repeated 10 times for each honey concentration.

## RESULTS

Comparison of the inhibition zones of 25 and 100% Dabur and manuka honey showed statistically significant differences. Independent sample t test was used to compare the means between groups.

**Fig. 1:**
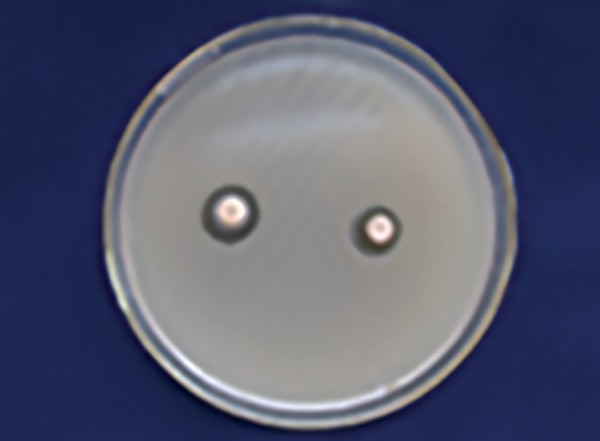
Dabur honey zone of inhibition 25% (S. *mutans)*

**Fig. 2:**
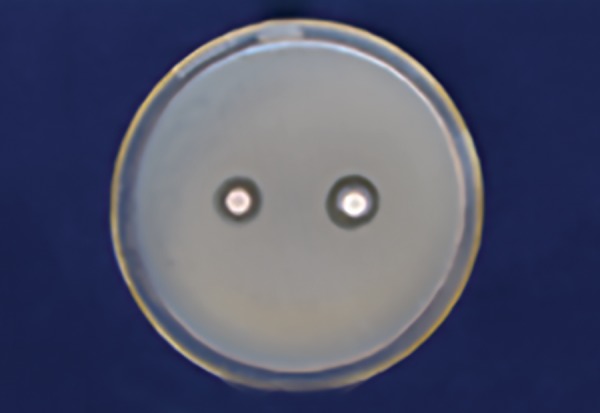
Manuka honey zone of inhibition 25% (S. *mutans)*

Table 1 shows that 25% Dabur honey (Fig. 1) has less inhibitory effect than 25% manuka honey in *S. mutans* zone (Fig. 2) with a significant p-value (p ≤ 0.001).

**Fig. 3:**
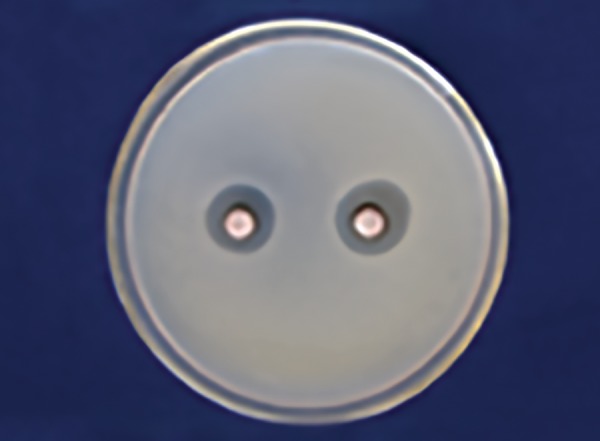
Dabur honey zone of inhibition 100% *(Lactobacillus)*

**Fig. 4:**
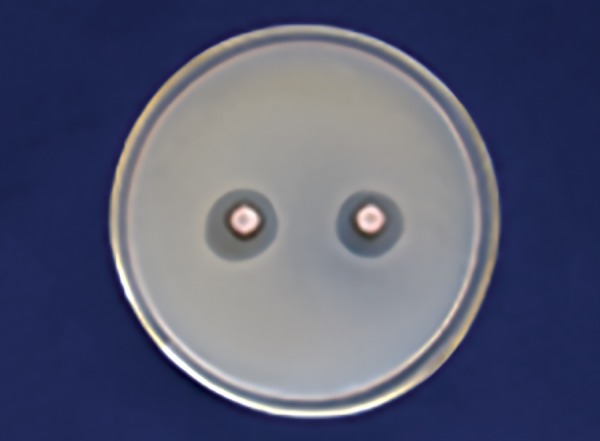
Manuka honey zone of inhibition 100% (S. *mutans)*

Table 2 shows that 100% of Dabur honey (Fig. 3) has less inhibitory effect than 100% manuka honey in *S. mutans* (Fig. 4) with a significant p-value (p ≤ 0.001).

**Table 1:** **Table 1:** Comparison of mean zone of inhibition for S. *mutans* between Dabur (25%) and manuka (25%) honey

*Organism*		*Honey*		*Mean*		*Standard deviation*		*Mean difference*		*t-value*		*p-value*	
*S. mutans*		Dabur 25%		9.8		1.5		–4.6		–7.48		<0.001 (Significant)	
		Manuka 25%		14.4		1.3							

**Table 2:** **Table 2:** Comparison of mean zone of inhibition for S. *mutans* between Dabur (100%) and manuka (100%) honey

*Organism*		*Honey*		*Mean*		*Standard deviation*		*Mean difference*		*t-value*		*p-value*	
*S. mutans*		Dabur100%		16.5		2.5		–4.7		–4.10		0.001	
		Manuka 100%		21.2		2.7							

**Table 3:** **Table 3:** Comparison of mean zone of inhibition for *Lactobacillus* between Dabur (25%) and manuka (25%) honey

*Organism*		*Honey*		*Mean*		*Standard deviation*		*Mean difference*		*t-value*		*p-value*	
*Lactobacillus*		Dabur 25%		10.9		1.2		–4.9		–8.71		<0.001	
		Manuka 25%		15.8		1.3							

**Fig. 5:**
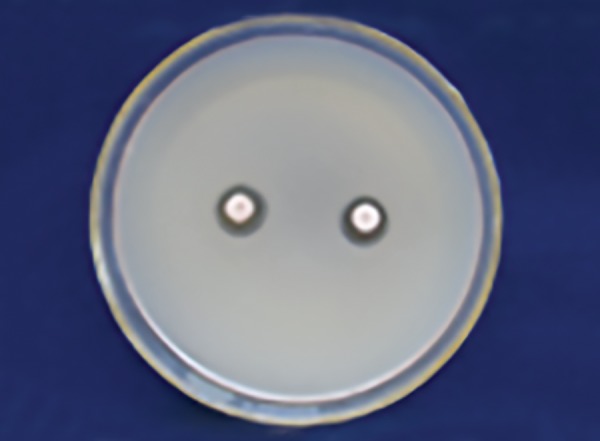
Dabur honey zone of inhibition 25% *(Lactobacillus)*

**Fig. 6:**
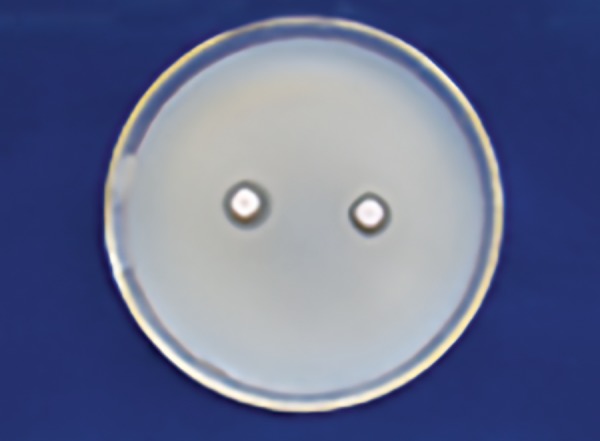
Manuka honey zone of inhibition 25% *(Lactobacillus)*

Table 3 shows that 25% Dabur honey (Fig. 5) has less inhibitory effect than 25% manuka honey in *Lactobacillus* zone (Fig. 6) with a significant p-value (p ≤ 0.001).

**Fig. 7:**
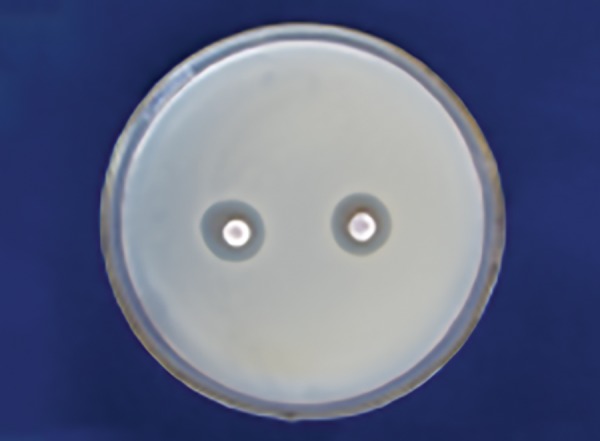
Dabur honey zone of inhibition 100% (S. *mutans)*

**Fig. 8:**
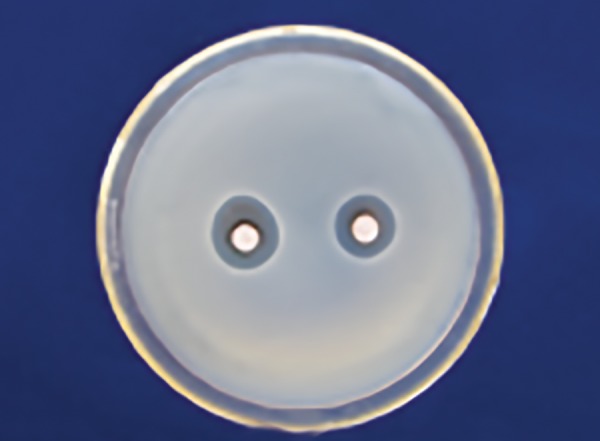
Manuka honey zone of inhibition 100% *(Lactobacillus)*

Table 4 shows that 100% of Dabur honey (Fig. 7) has less inhibitory effect than 100% manuka honey in *Lactobacillus* (Fig. 8) with a significant p-value (p ≤ 0.001).

Similarly, Tables 5 and 6 show that 25% of Dabur honey has less inhibitory zone than 100% of Dabur honey in *Lactobacillus* and *Streptococcus* zone with a statistical significance (p ≤ 0.001). Tables 7 and 8 show that 25% of manuka honey has less inhibitory zone than 100% of manuka honey in *Lactobacillus* and *Streptococcus* zone with a statistical significance (p ≤ 0.001).

## DISCUSSION

The antibacterial factors of honey are primarily due to enzymatic glucose oxidation reaction, high osmotic pressure, low water activity, acidic environment, high carbon-nitrogen ratio, low redox potential, phytochemicals, antioxidants,^14-16^ the hyperosmolarity effect (>80% sugar content), acidic pH, hydrogen peroxide, methylglyoxal, bee defensin-1, diverse proteinaceous compounds, flavo-noids, and phenolic compounds,^4,17,18^ but the foremost antimicrobial activity of most honeys is due to hydrogen peroxide.^19^

**Table 4:** **Table 4:** Comparison of mean zone of inhibition for *Lactobacillus* between Dabur (100%) and manuka (100%) honey

*Organism*		*Honey*		*Mean*		*Standard deviation*		*Mean difference*		*t-value*		*p-value*	
*Lactobacillus*		Dabur 100%		17.1		2.5		–5.1		–4.14		0.001	
		Manuka 100%		22.2		3.0							

**Table 5:** **Table 5:** Comparison of mean zone of inhibition for *S. mutans* between Dabur (25%) and Dabur (100%) honey

*Organism*		*Honey*		*Mean*		*Standard deviation*		*Mean difference*		*t-value*		*p-value*	
*S. mutans*		Dabur 25%		9.8		1.5		–6.7		–7.4		<0.001	
		Dabur 100%		16.5		2.5							

**Table 6:** **Table 6:** Comparison of mean zone of inhibition for *Lactobacillus* between Dabur (25%) and Dabur (100%) honey

*Organism*		*Honey*		*Mean*		*Standard deviation*		*Mean difference*		*t-value*		*p-value*	
*Lactobacillus*		Dabur 25%		10.9		1.2		–6.2		–7.1		0.001	
		Dabur100%		17.1		2.5							

**Table 7:** **Table 7:** Comparison of mean zone of inhibition for S. *mutans* between manuka (25%) and manuka (100%) honey

*Organism*		*Honey*		*Mean*		*Standard deviation*		*Mean difference*		*t-value*		*p-value*	
*S. mutans*		Manuka 25%		14.4		1.3		–6.8		–7.3		<0.001	
		Manuka 100%		21.2		2.7							

**Table 8:** **Table 8:** Comparison of mean zone of inhibition for *Lactobacillus* between manuka (25%) and manuka (100%) honey

*Organism*		*Honey*				*Mean*		*Standard deviation*		*Mean difference*		*t-value*		*p-value*	
*Lactobacillus*		Manuka		25%		15.8		1.3		–6.4		–6.2		0.001	
		Manuka		100%		22.2		3.0							

Manuka honey has a phytochemical component and a low hydrogen peroxide component. The nonperoxide antibacterial activity of typical manuka honey was tested against seven species of bacteria and compared with typical honey with a hydrogen peroxide component. The minimum inhibitory concentration (MIC) of honey was found to range from 1.8 to 10.8%.^20^ Types of honey differ greatly in their antimicrobial potency, varying as much as 100-fold. Manuka honey additionally contains d-gluconolactone, which reduces its pH and exerts antibacterial property. It also abundantly contains methyl syringate, ortho-methoxyacetophe-none, and 3-phenyl lactic acid, all of which inhibit bacterial growth.^21,22^

Methylglyoxal is particularly lethal toward bacterial growth by interrupting cell divisions, arresting growth, and specifically causing the degradation of bacterial deoxyribonucleic acid even at a very low concentration.^23^

Rupesh et al^6^ reported that manuka honey with UMF 15 is highly effective in reducing dental plaque and on the growth of cultures of oral bacteria.

Steinberg et al conducted study, on seven species of oral streptococci and found that the minimum inhibitory concentration of honey for *Streptococcus oralis* was 12%, for *Streptococcus anginosus* was 17%, and for *Streptococcus gordonii, Streptococcus mutans, Streptococcus salivarius, Streptococcus sanguis* was 25%.^22^.

English et al^23^ from their study concluded that the MIC of honey for *S. mutans* was 25% and for *Streptococcus sobrinus,* it was 35% and that the salivary bacterial count was reduced by 40% 1 hour after holding 5 mL of honey in the mouth for 4 minutes.

Mandal and Mandal,^14^ and Lin et al^15^ reported that honey inhibits the growth of a wide range of antibacterial activity on *S. mutans* and *Lactobacillus in vitro.* The results of this study are in concurrence with our study.

Oddo et al^24^ concluded that the use of a low harmful sweetener in the diet is very important, especially if it is confirmed that honey has antibacterial activity against cariogenic bacteria *in vitro* and *in vivo.^20^* The results of this study are similar to that of our study, which is statistically significant.

## CONCLUSION

Natural honey had an antibacterial activity on *S. mutans* and *Lactobacillus* bacteria. This effect depends on the concentration of honey used.

Summarizing the findings of our study, we would like to conclude that manuka honey is not as cariogenic as other sugars and has anticariogenic properties. Further research related to manuka honey as a sweetening agent can play a pivotal role in caries prevention, especially in children.

Further studies will be required to substantiate and propagate our preliminary observations.
